# *PTEN* hamartoma tumor syndrome: Clinical and genetic characterization in pediatric patients

**DOI:** 10.1007/s00381-024-06301-2

**Published:** 2024-02-26

**Authors:** Jesús Martín-Valbuena, Nerea Gestoso-Uzal, María Justel-Rodríguez, María Isidoro-García, Elena Marcos-Vadillo, Sandra Milagros Lorenzo-Hernández, M. Carla Criado-Muriel, Pablo Prieto-Matos

**Affiliations:** 1 Department of Pediatrics, University Hospital of Salamanca, Salamanca, Spain; 2 Institute for Biomedical Research of Salamanca, IBSAL, Salamanca, Spain; 3 Department of Medicine, University of Salamanca, Salamanca, Spain; 4 Clinical Biochemistry Department, University Hospital of Salamanca, Salamanca, Spain; 5 Department of Biomedical and Diagnostic Sciences, University of Salamanca, Salamanca, Spain

**Keywords:** Cowden syndrome, Macrocephaly, Children, Genetics

## Abstract

**Objective:**

The aim of this study was to provide a full characterization of a cohort of 11 pediatric patients diagnosed with *PTEN* hamartoma tumor syndrome (PHTS).

**Patients and methods:**

Eleven patients with genetic diagnostic of PHTS were recruited between February 2019 and April 2023. Clinical, imaging, demographic, and genetic data were retrospectively collected from their hospital medical history.

**Results:**

Regarding clinical manifestations, macrocephaly was the leading sign, present in all patients. Frontal bossing was the most frequent dysmorphism. Neurological issues were present in most patients. Dental malformations were described for the first time, being present in 27% of the patients. Brain MRI showed anomalies in 57% of the patients. No tumoral lesions were present at the time of the study. Regarding genetics, 72% of the alterations were in the tensin-type C2 domain of PTEN protein. We identified four *PTEN* genetic alterations for the first time.

**Conclusions:**

*PTEN* mutations appear with a wide variety of clinical signs and symptoms, sometimes associated with phenotypes which do not fit classical clinical diagnostic criteria for PHTS. We recommend carrying out a genetic study to establish an early diagnosis in children with significant macrocephaly. This facilitates personalized monitoring and enables anticipation of potential PHTS-related complications.

## Introduction

*PTEN* hamartoma tumor syndrome (PHTS) is a disease with a broad spectrum of signs and symptoms. First described in 1993, it includes the classical Cowden syndrome (CS), Bannayan-Riley-Ruvalcaba syndrome (BRRS), Lhermitte-Duclos disease (LDD), and Proteus and Proteus-like syndrome. The phenotypic spectrum of PHTS has been evolving and expanding paralleling the increasing accessibility of genetic diagnosis. There are currently many other alterations related to *PTEN* pathogenic variants such as neurodevelopmental disorders, segmentary overgrowth, autistic spectrum disorder (ASD), or macrocephaly. In the pediatric population, classic forms with mucocutaneous manifestations, hamartomatous lesions, and malignancies are less common than development delay, brain magnetic resonance imaging (MRI) alterations, and growth disorders such as macrocephaly, overweight, or limb asymmetries [1]. The etiology of PHTS lies with the *PTEN* (phosphatase and tensin homolog deleted on chromosome 10) gene. The protein encoded by this gene is a dual phosphatase with both lipid and protein activity. It is ubiquitously expressed in different cells. As regards the lipid activity, it dephosphorylates phosphatidylinositol-3, 4, 5-phosphate (PIP3), a mediator in the MAPK pathway. This action induces cell cycle arrest in G1 and apoptosis, regulating cell growth. It is known as a tumor suppressor, mutated in different cancer types. Moreover, it has been associated with insulin regulation pathways and mitochondrial metabolism [2]. Pathogenic variants of this gene have been reported to cause PHTS. The exact prevalence of PHTS is unknown due to the high variability in the manifestations and the difficulties to carry out massive genetic testing. The prevalence of CS was estimated in 1:250,000 in the Dutch population, although the global real prevalence of PHTS is likely to be higher [3].

Different types of genetic variants have been reported that affect the *PTEN* gene without a clear genotype-phenotype correlation. An apparent lack of missense mutations has been described in LDD [3]. However, missense variant 5′ to or within the phosphate core motif has been associated with involvement of five or more organs, although none of these genotype-phenotype correlations has been confirmed in large case series [4]. *PTEN* pathogenic variants include missense and nonsense nucleotide variants, deletions, insertions, and splicing mutations, all of them with autosomal dominant inheritance [5]. Frequency of de novo mutations is estimated to be between 10.7 and 47.6% [6]. Mosaicism for *PTEN* has also been described in PHTS.

The penetrance of PHTS is near 100% by the fourth decade in patients with a pathogenic variant in *PTEN* [2]. The clinical manifestations can vary between different individuals: macrocephaly and neurodevelopmental disorders are more frequent in childhood while classical symptoms (intestinal polyps, malignancies) are more frequent in patients diagnosed in adulthood without a clear evolution from one spectrum to the other.

The literature regarding PHTS in children is limited. The largest series of cases published in Pubmed includes just sixteen cases, so we aim to describe a cohort of eleven children with a pathogenic variant of *PTEN*, diagnosed in the “Reference Unit of Rare Diseases Advanced Diagnosis” (DiERCyL) to provide more data to the literature detailing the clinical, genetic, and other issues related to this condition.

## Patients and methods

This study includes patients diagnosed with PHTS between February 2019 and April 2023 in DiERCyL. Demographic, clinical, and genetic data were recorded retrospectively from their hospital medical history.

### Genetic studies

The genetic studies were carried out in the Laboratory of Molecular Genetics and Pharmacogenetics at University Hospital of Salamanca. Genomic DNA was extracted from peripheral blood leukocytes by magnetics beads or silica-membrane-based nucleic acid purification. Whole exome sequencing was performed on the NextSeq 500 platform (Illumina; San Diego, CA). For this purpose, DNA libraries were prepared using TruSeq technology (Illumina; San Diego, CA) and captured using xGen Exome technology (Integrated DNA Technologies, IDT; Coralville, IA). A bioinformatic study of the DNA sequences obtained was performed by comparison with the reference genome version GRCh37/hg19. The genetic variants detected were confirmed by Sanger sequencing. The assessment of the pathogenicity of the variants was determined according to the American College of Medical Genetics and Genomics (ACMG) guidelines, using the ClinVar database and Varsome scores. Genetic studies were amplified to parents and siblings, when possible (six out of eleven index cases), although they do not present clinical features of PHTS.

### Signs and symptoms

According to clinical signs, macrocephaly was defined as percentile > 97 and standard deviation (SD) was calculated according to the anthropometric standards published for the Spanish population [7]. Height and weight percentiles and SD were calculated according to Spanish growth studies from 2010 [8]. In case it was required, a clinical examination by a pediatric neurologist was performed in order to evaluate ASD and development delay (DD). MRI was performed in all but four patients. Other complementary tests and oncologic follow-up were individualized according to the clinical spectrum and the individual risk.

## Results

### Clinical and demographic data

As summarized in Table 1, we have studied 11 patients, 7 males and 4 females, diagnosed with PHTS. The mean age was 6.87 ± 3.02 (1.91–11.5) years. In our cohort, we have two siblings with the same mutation.

**Table 1:** Demographic and clinical data as well as the reason for derivation to genetic study of the patients

**Patient**	**Gender**	**Age**	**Height (cm)**	**Weight (kg)**	**Diagnosis**	**Referring clinician**	**DD**	**DD improvement**	**ASD**	**Macrocephaly**	**Facial features**	J**oint laxity**	**Skin**	**Tonsillar hypertrophy**	**MRI**
**1**	Male	3 years 5 months	106 +1.53 SD	19 +1.15 SD	DD, macrocephaly, dysmorphic features	Pediatric neurologist	Motor and language	Yes	Yes	Yes +4.3 SD	Bossed forehead, prognathism	Yes	Café-au-lait macules, thumb hamartoma	-	-
**2**	Male	9 years 2 months	143 +0.88 SD	60.5 +3 SD	Macrocephaly, overweight	Pediatric neurologist	Motor	Yes	Yes	Yes +5 SD	-	-	Unknown	-	Normal
**3**	Male	10 years 5 months	158 − 0.8 SD	48.8 − 0.74 SD	Macrocephaly	Pediatric neurologist	Motor	Yes	No	Yes	Acrocephaly, bossed forehead, prognathism	No	Café-au-lait macules, keloid scar, follicular keratosis	No	Prominent perivascular spaces
**4**	Female	9 years 2 months	134.8 − 0.07 SD	40 +0.94 SD	Intentional tremor	Pediatric neurologist	No	-	No	Yes +5.08 SD	Bossed forehead, prognathism, dental agenesis, low-set ears, hypertelorism	No	Cervical lipoma	No	Prominent perivascular spaces, tonsillar descent
**5**	Male	11 years 6 months	-	-	Euthyroid multinodular goiter	Pediatric endocrinologist	No	-	No	Yes +2.6 SD	-	No	Unknown	No	-
**6**	Female	8 years 9 months	147 +2.36 SD	39.5 +1.14 SD	DD, dysmorphic features, overheight, central hypotonia diffuse, leukoencephalopathy	Pediatric neurologist	Motor and language	Yes	No	Yes +4.68 SD	Hypomimia, dental agenesis, low-set ears, hirsutism, stellate iris	Yes	No	Yes	Diffuse leukoencephalopathy
**7**	Female	7 years 3 months	Overheight (no data)	-	Macrocephaly, overheight	Pediatric neurologist and pediatric endocrinologist	Motor and language	-	No	Yes +4.58 SD	Hypertelorism, dental malocclusion, gingival hyperplasia	No	No	Yes	Normal
**8**	Female	5 years 8 months	-	-	LDD	Pediatric neurologist	No	-	No	Yes	-	No	No	Yes	Cortical cerebellar dysplasia (LDD)
**9**	Male	4 years 4 months	-	-	DD, macrocephaly, hypercholesterolemia	Pediatric neurologist	Motor and language	No	No	Yes +3.89 SD	Normal	No	No	Yes	Normal
**10**	Male	4 years 0 months	-	17.5 +0.03 SD	DD, macrocephaly, hypotonia, dysmorphic features	Pediatric neurologist	Motor and language	Yes	Yes	Yes +4.22 SD	Coarse facial features, hypertelorism, low-set ears, wide nasal bridge, bossed forehead	No	No	No	-
**11**	Male	1 year 11 months	87 0 SD	15.3 +1.85 SD	DD, macrocephaly	Pediatric neurologist	Motor and language	No	No	Yes +4.37 SD	Bossed forehead	Yes	No	No	-

The age at diagnosis ranged from 1 year and 11 months to 11 years and 6 months. All genetic studies except one were requested by a pediatric neurologist, while the other one was requested by a pediatric endocrinologist. The most prevalent diagnoses prior to the genetic study were macrocephaly (11/11), developmental delay (5/11), and overgrowth (3/11). The mean head circumference SD score was +4.3 ranging from +2.6 to +5. According to their growth, three of our patients were diagnosed with some kind of overgrowth. One of them presented overweight (+3 SD), another one overheight (+2.35 SD), and the last one was diagnosed with overheight with no data available about her height.

In general, our patients did not present facial dysmorphism, and most of the anomalies were secondary to macrocephaly. Facial phenotype was not described in two of the patients. In the rest of them, frontal bossing (5/9), prognathism (3/9), and dental anomalies such as dental agenesis or dental malocclusion (3/9) were the most frequent phenotypic manifestations. No facial asymmetries were reported, although two of the patients showed slight asymmetry of the lower limbs.

Cutaneous signs were observed in three of the patients. Patient 1 had cafe-au-lait macules and a hamartoma on the thumb. Patient 2 also showed cafe-au-lait macules besides follicular keratosis and a keloid scar. Patient 3 had had a cervical lipoma removed. No other dermatological findings were described. Three of our patients showed joint laxity without presenting joint pain, dislocations, Marfan phenotype, or other manifestations related to collagen disturbance such as varicose veins, hernias, or uterine/anal prolapse. Four of the patients presented tonsillar hypertrophy, one of them requiring surgical removal due to recurrent tonsillitis and sleep apnea/hypopnea syndrome.

Seven of our patients (64%) suffered some kind of developmental delay, and five of them (71%) presented clinical improvement in their symptomatology. Our cohort showed a wide range of neuropsychiatric involvement. Six patients showed both speech and motor delay and two patients showed only motor delay. ASD was reported in three patients (27%), the rest showed normal social behavior. Specific intelligence quotient tests were not performed.

Currently, none of our patients has developed any malignancies, although they are still young and oncologic screening is being conducted. Only patient 5 has thyroid involvement, with multinodular goiter, and he is waiting for surgical removal due to mild dysphagia and aesthetic reasons. A special case is patient 4, with a diagnosis of intention tremor and macrocephaly without any other neurological symptoms. This patient’s phenotype included frontal bossing, prognathism, dental agenesis, short limbs and fingers, low-set ears, short philtrum, low nasal bridge, and hypertelorism. She had a cervical lipoma removed prior to diagnosis. As far as we know, none of these signs has been described in PHTS. Brain MRI showed an enlargement of perivascular spaces and a 2-mm tonsillar descent. A de novo nonsense mutation in *PTEN* gene was detected during the genetic study.

### Imaging studies

A brain MRI was performed in seven of our patients following symptomatic criteria. Three of the patients (43%) showed no abnormalities in MRI. Two patients presented enlarged perivascular spaces, one of them with a 2-mm tonsillar descent. Another patient was diagnosed with diffuse leukoencephalopathy and patient 8 showed cerebellar asymmetry and cortical dysplasia compatible with LDD.

### Genetic studies

Genetic analysis was performed to all the patients, and Sanger sequencing or confirmed nine heterozygous pathogenic mutations, three of them not described before up to our knowledge. The most common mutation was nonsense point mutation, identified in six patients (55%). Two of the patients presented missense point mutation and the other two frameshift insertion. In patient 10, a deletion involving the region of the *PTEN* gene was found by CGH-arrays and confirmed by quantitative PCR.

**Fig. 1:**
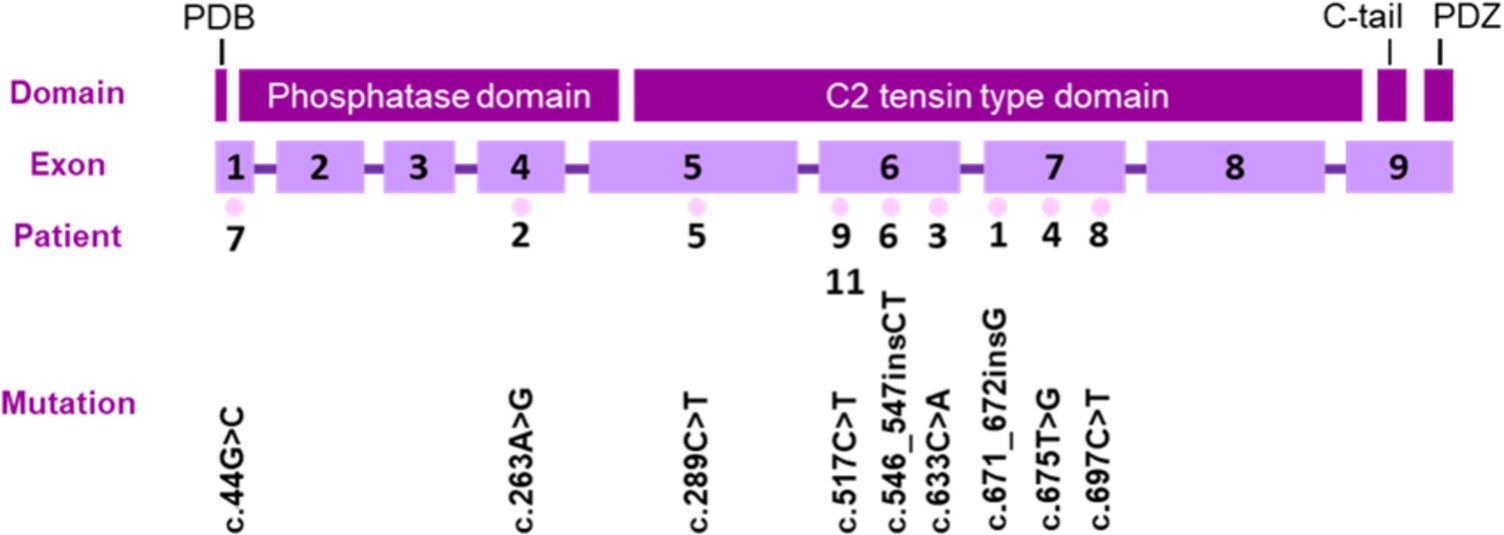
*PTEN* structure and location of the mutations detected

Table 2 contains the genetic data of our patients. We were not able to establish a clear genotype-phenotype correlation because of the sample size, although the two missense mutations were in a hotspot region according to VarSome. This region includes a phosphatase domain that contains the CX5R signature motif for phosphatases [2]. One of our patients presented a deletion involving part of the *PTEN* gene. The rest of our patients’ mutations affect the tensin-type C2 domain and all of them are point nonsense mutations except for one frameshift insertion (patient 6). The mutation detected in patient 3 has been reported to generate a truncated protein affecting the functionality of a C-terminal C2 domain with different phenotype expressions [9–11]. Mutation in patient 8 affects a putative tyrosine phosphatase domain affecting the tertiary structure of the protein [12, 13]. The molecular effect of the rest of the mutations detected has not been described for the moment. Figure 1 shows the *PTEN* structure and the location of the detected mutations.

**Table 2:** Genetic alterations detected in the patients. Mutation location was determined using the mRNA reference sequence NM_000314.8. Hotspot region refers to areas of DNA which are more likely to mutate according to the information from Uniprot obtained in VarSome platform

**Patient**	**Type of alteration**	**Mutation location**	**Aminoacid change**	**Hotspot region**	**ClinVar classification**	**Varsome score**	**Reported**
**1**	Insertion-frameshift	c.671_672insG	p.Ile224MetfsTer19	Yes	Not reported	Likely pathogenic	No
**2**	Missense point mutation	c.263A>G	p.Tyr88Cys	Yes	Not reported	Uncertain significance	No
**3**	Nonsense point mutation	c.633C>A	p.Cys211Ter	No	Pathogenic	Pathogenic	Yes (PS, BRR, CS)
**4**	Nonsense point mutation	c.675 T>G	p.Tyr225Ter	No	Pathogenic	Pathogenic	Yes
**5**	Nonsense point mutation	c.289C>T	p.Gln97Ter	No	Pathogenic	Pathogenic	Yes (LDD, macrocephaly, skin pathology, intestinal polyps)
**6**	Insertion-frameshift	c.546_547insCT	p.Lys183LeufsTer17	Yes	Not reported	Likely pathogenic	No
**7**	Missense point mutation	c.44G>C	p.Arg15Thr	Yes	Likely pathogenic	Pathogenic	Yes
**8**	Nonsense point mutation	c.697C>T	p.Arg233Ter	No	Pathogenic	Pathogenic	Yes (macrocephaly, gynecomastia, trichilemmoma, multinodular goiter)
**9**	Nonsense point mutation	c.517C>T	p.Arg173Cys	Yes	Pathogenic	Pathogenic	Yes
**10**	Deletion	Del10q23.1-q23.31	NA	NA	NA	NA	No
**11**	Nonsense point mutation	c.517C>T	p.Arg173Cys	Yes	Pathogenic	Pathogenic	Yes

Segregation studies were performed in six of our patients, including both parents in four of them. The mutation found in the siblings (patient 9 and patient 11) was also present in their mother, suggesting maternal inheritance, although for the moment she does not present any symptoms or oncological history. Moreover, a de novo mutation was confirmed in two patients. Finally, in patients 1 and 2, just the father or the mother were studied, and no *PTEN* mutations were found.

## Discussion

PHTS involves a broad clinical spectrum, where the oncologic risk is the main point under consideration. There is not much literature on this syndrome in the pediatric population. Through this retrospective study, we try to offer new data according to clinical, genetic, and neuroimaging findings in our PHTS cohort.

The molecular approach to certain pathologies has made it possible to know the underlying cause behind many of them. On some occasions, a known disease or syndrome has been subclassified into several different pathologies according to their molecular substrate. In other cases, such as PHTS, a variety of different syndromes (e.g., CS, BRRS, PS…) have shown a common etiology, in this case, alterations in the *PTEN* gene affecting its protein functionality. PHTS has been widely studied in adults due to its tendency to develop different kinds of tumors. However, it has barely been described in the pediatric population. We aim to offer new data in pediatric patients and to correlate our patients with the existing literature. In our study, we have identified four new *PTEN* alterations that need to be considered when studying PHTS.

There are no differences in prevalence associated to sex described in the literature. Several series of cases with PHTS show male preponderance while others do not show any sex predisposition [5, 14–16]. In our cohort of 11 patients, seven of them are male. As developmental disorder is more common in males and it was the leading sign in most of our patients, this might be associated with our findings [17].

As shown in the “Results” section, we were not able to establish a clear genotype-phenotype correlation according to previous studies, including a review of the clinical literature [18]. However, it has been hypothesized after considering some facts. For example, there are no data about missense variants detected in LDD disease in the literature, all of them are caused by a nonsense or frameshift variant which is in line with the clinical and genetic data of patient 8 [3]. It has been suggested that pathogenic variants 5′ to or within the phosphatase core are associated with higher severity and involvement of five or more organs; however, these studies are based on an adult population, and no conclusions can be established in our cohort, although a narrower oncologic screening may be implemented in these patients [1]. There is one study which hypothesizes about a correlation between the phenotype and post-transcriptional *PTEN* expression and its splice variants. However, we cannot add new data because none of our mutations was in the promoter region and no transcriptional studies were conducted [9]. A more recent study using an artificial humanized yeast model and evaluating lipid phosphatase activity in different *PTEN* mutations showed a higher phosphatase activity in ASD-associated variants [19]. This fact, albeit interesting, is difficult to evaluate in real patients, but it opens a window for future research and better understanding of the PHTS.

Macrocephaly is a major sign in PHTS. All our patients presented macrocephaly, as it has been reported in several other cohorts [5, 20]. Seventy-eight percent of our sample (7/9) had a head circumference SD score over + 4, greater than a French cohort and similar to an Italian cohort, which are the studies closest to the Spanish population [5, 20].

According to a *PTEN* review in childhood, the most common facial characteristics described were frontal bossing, depression of nasal bridge, horizontal eyebrows, and dolichocephaly [9]. Frontal bossing was present in approximately half of our patients, which could be secondary to the macrocephaly. Other common features described in the literature were less frequent in our patients. Only two patients presented depression of the nasal bridge and one patient showed acrocephaly, although dolichocephaly has been reported in other series [21]. Prognathism was described in three of our patients while we have just found one case in the literature reporting this sign [22]. It is also remarkable that teeth alterations such as agenesis and malocclusion were present in three of the patients. This fact has not been described up to our knowledge and could be secondary to gingival disturbances which are more common, but it is also possible that *PTEN* mutations imply dental issues because *PTEN* plays an important role in osteogenesis and proliferation in dental pulp cells [18, 23].

Neuropsychiatric involvement has also been described in the literature, with a wide spectrum of signs including motor and speech delay, ASD, and intellectual disability which tends to improve, although some alterations persist through adulthood [5, 18]. Seven of our patients (64%) presented some grade of developmental delay, which was only motor in two of them and motor and language-related in the rest. This prevalence is lower than the prevalence reported in other case series and comparable to findings from an Italian study [5, 21]. Four of the affected patients showed an improvement in successive controls and in two of the patients who did not present neurological improvement this might be due to a short follow-up period. Developmental impairment during childhood with a subsequent normal cognitive outcome in adulthood has been previously reported [24].

ASD is another of the main neuropsychiatric issues related to *PTEN* alterations. Three of our patients (27%) had some symptoms of the autistic spectrum. These data are similar to those of other studies suggesting a strong association between *PTEN* and ASD [5]. The underlying cause could be an alteration in neuronal growth, survival, and migration via the PI3K/AKT pathway as well as a synergy between *PTEN* mutations and other alterations in autism susceptibility genes [25].

There are several manifestations described in brain MRI in PHTS. However, it is difficult to assess accurately the prevalence of these alterations, especially in the pediatric population since MRI is not a diagnostic test exempt from certain risk. In general terms, it was just conducted in those patients who showed neuropsychiatric signs. In our series, brain MRI was performed in seven patients, and three were completely normal (43%) which is a greater percentage than reported in other studies, especially considering that, in the Italian cohort, MRI was performed in all patients, including those without neurological involvement [5, 15]. Enlarged perivascular spaces were found in 29% of our patients, which was less frequent than the 94% prevalence previously described [15]. In this study, 36% of their patients presented white matter abnormalities, defined as changes in signal intensity. This condition was detected in only one of our patients. One of our patients presented a tonsillar descent of 2 mm, which represents a lower prevalence than the 33–37% reported by other studies involving adults and children, respectively [5, 26]. LDD is difficult to assess in children due to the lack of the typical “tiger-stripe” in T2. One of our patients presented cortical cerebellar dysplasia compatible with LDD. Despite being a pathognomonic criterion for CS in adults, this finding has not been reported in several case series in children’s populations with PHTS and the relation between LDD and PHTS in pediatric populations remains unclear according to PHTS reviews in children, although in some serial of cases of pediatric patients with LDD, some of them meet CS clinical criteria [18].

Vascular features such as hemangiomas, cavernomas, or arteriovenous malformations, which are a major sign in several of the classic syndromes related to *PTEN* alterations (CS, BRRS…), have not been reported in our cohort, although they may appear later in the natural evolution of the disease [18].

*PTEN* alterations have been frequently associated with skin features, which were a CS criterion even before the discovery of the *PTEN* gene. Hamartomatous growths of the same or different tissues (e.g., trichilemmomas, fibromas, lipomas) can be detected in almost every part of the body, although they tend to appear on the face and surrounding orifices [5, 18]. Acral keratoses and tongue alterations are some of the most prevalent skin alterations in PHTS [18]. The presence of several of these features, especially in a macrocephalic child, is very suggestive of PHTS [5]. In our cohort, one patient presented a hamartoma on the thumb and another patient presented a cervical lipoma. The prevalence is lower than what has been reported in other studies, but this may be due to the age-related penetrance described in skin lesions [5, 18]. Two of our patients presented cafe-au-lait macules, which is not a typical manifestation of PHTS, and its prevalence has not been studied for the moment, although there are several case reports which include this alteration [27−29]. One of these patients also presented keloid scarring. The *PTEN* gene may play an important role in keloid scarring as suggested in a case control study that demonstrates underexpression of *PTEN* in keloid samples compared to normal controls [30]. Penile freckling is another classic hallmark of PHTS, especially in the pediatric population. It has been reported in childhood from the first age of life and was absent in our cohort [18].

One of the main concerns of PHTS is the oncological risk. *PTEN* mutations have been associated with thyroid, breast, endometrium, and kidney tumor, among others that are less frequent [1]. Defining the exact risk of developing malignancies is difficult. Studies involving CS, BRRS, or PS patients prior to molecular diagnosis had an important recruitment bias. In our cohort, many of the new diagnosed children were just studied because of macrocephaly and developmental problems. There is a lack of longitudinal studies in this group of patients to accurately estimate oncological risk. Although some malignant tumors have been reported in children, such as thyroid and renal cell carcinoma, granulosa cell tumor of the ovary, or colonic ganglioneuroma, none of our patients has presented any malignancy for the moment [31]. This lack of evidence in the literature makes it difficult to establish the best clinical management of these children. A 2019 review of PHTS in children proposes several management considerations for these patients, considering that in spite of the lack of data about the prevalence of oncologic complications in children, there have been some cases reported [18, 31]. Achieving an early diagnosis through a *PTEN* genetic study in pediatric patients with macrocephaly would allow an early diagnosis of possible malignancies by performing an accurate follow-up of these patients.

## Conclusions

This study shows the wide variety of clinical signs and symptoms associated with *PTEN* mutations, which sometimes express phenotypes which do not meet any of the classic diagnostic criteria for CS.

All except one of our patients were referred by a pediatric neurologist, and macrocephaly and neurodevelopmental issues were the main reason to initiate genetic studies. We highly recommend looking for *PTEN* mutations in children with pronounced macrocephaly, especially if they present other symptoms such as neurodevelopmental disorders, ASD, certain facial dysmorphisms, or thyroid nodules.

In PHTS, as in other heterogenous syndromes, it is important to describe as many clinical manifestations as possible in order to get a better knowledge of the disorder and help other clinicians to reach an early diagnosis. Being able to diagnose PHTS during childhood makes it possible to keep a closer follow-up in the patients and detect certain complications associated earlier, improving the treatment and, subsequently, the prognosis and the quality of life. This is especially relevant in oncologic issues, in which an individualized screening for malignancies may be useful to detect tumors in earlier stages. Besides, as it is a hereditary syndrome, genetic counselling could be possible with an early diagnosis.

Molecular diagnosis may be difficult to carry out in many centers. Whole exome sequencing usually takes too much time and resources to get accurately to a diagnosis. That is why being able to direct the molecular study according to the clinic features will be very helpful to save both money and time.

## Data Availability

Sequencing raw data is stored by the Reference Unit of Rare Diseases Advanced Diagnosis of Castile and Leon (DiERCyL). The clinical data mentioned in the manuscript was obtained from the hospital medical history of the patients.
